# Design, Implement, Sustain: Advancing Program‐Led Interventions for Binge Eating, a Narrative Review

**DOI:** 10.1002/erv.70118

**Published:** 2026-04-16

**Authors:** Bernou Melisse, Sanne F. W. Marcic‐van Doornik, Annemarie van Elburg

**Affiliations:** ^1^ Co‐Eur Utrecht the Netherlands; ^2^ American Center for Psychiatry and Neurology Abu Dhabi Abu Dhabi UAE; ^3^ Department of Clinical Psychology Utrecht University Utrecht the Netherlands; ^4^ Department of Medical and Clinical Psychology Tilburg University Tilburg the Netherlands; ^5^ Department of Clinical Psychology and Experimental Psychopathology University of Groningen Groningen the Netherlands

**Keywords:** eating disorders, effectiveness, implementation, program‐led interventions, stepped care

## Abstract

**Objective:**

Most individuals with binge eating never receive treatment, partly due to a lack of specialists and long waitlists. Program‐led interventions are scalable, and their therapeutic content and guidance are embedded within the program itself. However, they are not widely implemented in routine care. Strategies are proposed to overcome key barriers, to enhance the implementation, adherence, and reach of program‐led interventions in routine care for binge eating.

**Methods:**

Recent evidence from a structured keyword‐based search on program‐led interventions for recurrent binge eating is synthesised, and strategies are proposed to optimise their design, personalisation, and implementation.

**Results:**

Successful implementation of program‐led interventions depends on intervention format, support, and user characteristics. Specialist‐guided models reduce dropout rates, enhance adherence and acceptance, and support monitoring of adverse events. Brief, personalised digital interventions with synchronised therapist–user contact may reduce dropout probability and increase user and therapist adherence. Training and supervision promote therapist adherence and acceptance within routine care.

**Conclusion:**

Program‐led interventions offer scalable, cost‐effective care, but require balance between guidance, automation, contextual fit, and scalability. Challenges include resource demands, technological infrastructure, and applicability across diverse populations. Future research should test hybrid and algorithmically guided strategies, assess feasibility, equity, safeguard safety and privacy to support sustainable implementation.

## Highlights

- Program‐led interventions for binge eating can improve access to specialised treatment and reduce waitlists.
- Program‐led interventions are scalable and (cost‐) effective, but depend on thoughtful implementation, therapist training, supervision, and infrastructure.
- Interventions that are specialist‐guided, digital, brief, and personalised reduce dropout rates and increase adherence and acceptance.

## 1 Background

### 1.1 The Unmet Need for Accessible Treatment for Binge Eating

The majority of individuals with an eating disorder never receive a formal diagnosis or evidence‐based treatment (Ali et al. 2025; Bray et al. 2022), especially individuals with binge‐eating (Bray et al. 2022), non‐restricting‐ pathology, and higher body weights (Richards et al. 2022). Reasons for not receiving treatment include lack of therapists, long waitlists, financial costs, geographical barriers, not knowing where to seek help, and stigma associated with seeking help (Dalle Grave 2025; Johns et al. 2019; Linardon et al. 2020). Delayed access to treatment is associated with psychological distress, struggle with academic and professional attainment, and reduced chances of recovery (Allen et al. 2023; O. Carter et al. 2012; Flynn et al. 2021). These findings underscore the critical need for accessible, effective, and scalable interventions (Davey et al. 2023).

### 1.2 Program‐Led Interventions: A Scalable Cognitive Behaviour Therapy‐Based Approach

In the context of the need for scalable and accessible interventions, program‐led interventions are considered low‐intensity treatments, requiring less therapeutic input than conventional, high‐intensity, therapist‐delivered protocols, and are not simply ‘non–in person’ formats (Davey et al. 2023; Shafran et al. 2021). Program‐led interventions are cognitive behaviour therapy (CBT)‐based interventions in which therapeutic content and guidance are embedded within the program itself and delivered by self‐help materials or digital platforms, rather than being primarily delivered or adapted by a therapist (Davey et al. 2023). In other words, the defining feature is program‐embedded guidance and not merely reduced therapist contact time. Program‐led interventions can be offered in paper‐pencil (Fairburn 2013; Striegel‐Moore et al. 2010), digitalised, unguided (Murphy et al. 2024; Rohrbach, Dingemans, Spinhoven, et al. 2022), and guided formats (Melisse, Blankers, et al. 2023; Melisse, Blankers, et al. 2023). Guidance can be provided by a specialist or a non‐specialist (Shafran et al. 2009, 2021). In addition, therapist guidance can be synchronised (e.g., through video‐conferencing) or non‐synchronised (e.g., through messaging; ter Huurne et al. 2015). Furthermore, digitalised interventions can be interactive (e.g., video tutorials and graphic texts) or only offered in written text (Linardon et al. 2022).

### 1.3 Effectiveness, Cost‐Effectiveness, and Dropout

A recent meta‐analysis shows that CBT is effective for program‐led (guided self‐help and unguided self‐help) formats (Cuijpers et al. 2025), particularly for recurrent binge eating, including binge‐eating disorder and bulimia nervosa (Cruz et al. 2025; Cuijpers et al. 2025). There is, however, insufficient evidence for anorexia nervosa (Ambwani et al. 2014), hence the present narrative review focuses on program‐led interventions for recurrent binge eating (Cuijpers et al. 2025). After following program‐led interventions, in randomised controlled trials (RCTs), up to 58% of individuals with binge‐eating disorder report abstinence from binge eating, and up to 80% clinically benefitted (Eating Disorder Examination‐Questionnaire scores below the clinical cut‐off (Fairburn and Peveler 1990; Hilbert et al. 2020; Melisse, Blankers, et al. 2023; Wagner et al. 2013). This is 83% and 29% respectively, in the few implementation papers published (Melisse, Blankers, et al. 2023; Rom et al. 2023). Eating disorder pathology is reduced with a medium to large effect sizes (Hilbert et al. 2019, 2020). The efficacy of program‐led interventions is suggested to be on par with conventionally delivered evidence based treatments (van Beers et al. 2025). In addition, program‐led interventions generally incur lower costs than conventionally delivered treatments. Guided formats for binge‐eating disorder, for example, cost €630–900 in total or €18 per binge‐eating episode prevented, compared to about €3500 in total for conventionally delivered treatment (Jenkins et al. 2021; König et al. 2018; Melisse, Blankers, et al. 2023). Conversely, the cost‐efficiency ratio of unguided interventions is considered low given the high dropout rates (Beintner et al. 2014; Linardon et al. 2022). Dropout rates are generally higher compared to conventionally delivered interventions, especially in unguided and longer formats, with dropout rates ranging from 1% to 88% depending on delivery mode and the level of guidance (Linardon and Fuller‐Tyszkiewicz 2020; Linardon et al. 2022).

### 1.4 Bridging the Practice Gap: Optimising Implementation

The present narrative review addresses the current practice gap by exploring how to optimise the implementation and dissemination of program‐led interventions for eating disorders characterised by recurrent binge eating in order to increase their implementation, adherence, reach, acceptability, and therefore clinical impact. The present narrative review synthesises potential solutions to key barriers of implementation, including four domains: low dropout probability, adherence, acceptance, and safety and risk monitoring. Evidence‐informed principles for sustainable dissemination are proposed. The majority of the practical solutions outlined are drawn from existing empirical studies and a few implementation reports. However, the novelty and added value of the present narrative review lies in integrating these implications together for clinicians, researchers, and policymakers in a focused synthesis of actionable implementation strategies for CBT‐based program‐led interventions in routine clinical care. The present review is based on a structured search strategy, but not intended as a comprehensive systematic synthesis of all scalable treatment formats. The aim of the current narrative review is to bridge the gap between research and routine care by synthesising practical strategies, to optimise the effectiveness and implementation of program‐led interventions for recurrent binge eating.

## 2 Search Strategy and Study Selection

Peer reviewed studies in English were included from 2000 onwards. The literature was systematically searched and reviewed from PubMed, PsycINFO, and Web of Science. The search aimed to identify studies that report on CBT‐based program‐led interventions for recurrent binge eating. To facilitate this, a list of keywords related to eating disorders and intervention format (binge eating, binge‐eating disorder, bulimia nervosa, cognitive behaviour therapy, cognitive behavioural therapy, CBT, guided self‐help, self‐help, computerised, internet‐based, web‐based, digital, eHealth, telehealth, program‐led, stepped care) was used. To identify additional studies, reference lists of theses studies were screened. Top be included, studies (a) evaluated a CBT‐based program‐led intervention in which therapeutic content and guidance were embedded in the program, (b) targeted recurrent binge eating (e.g., binge‐eating disorder or bulimia nervosa), and (c) reported clinical or implementation‐relevant outcomes in clinical or routine‐care settings. The first author independently titles and abstracts, uncertainties were resolved through discussion.

## 3 Approaches to Address the Practice Gap

### 3.1 Dropout Probability

#### 3.1.1 Intervention Characteristics

Treatment dropout refers to the premature termination of the intervention before completing the recommended course, potentially impacting treatment outcomes, whereas study dropout refers to non‐completion of the assessments, regardless of treatment completion (Matthews et al. 2004). Based on the reviewed literature, Table 1 shows that treatment dropout rates can be reduced by offering digitalised, interactive interventions (Linardon et al. 2022), guided formats (J. C. Carter and Fairburn 1998; Grilo et al. 2005; Striegel‐Moore et al. 2010; ter Huurne et al. 2017), with synchronised contact between therapist and user (König et al. 2018; Melisse, Blankers, et al. 2023; Melisse, Blankers, et al. 2023; Puls et al. 2020). In addition, longer interventions experienced higher treatment dropout rates compared to more brief interventions (Beintner et al. 2014). Effective program‐led interventions were also characterised by flexibility and responsiveness. A review reported a U‐shaped pattern in treatment dropout rates, with user dropout probability occurring more frequently in individuals with mild symptoms due to recovery or in individuals with severe symptoms due to disengagement (ter Huurne et al. 2017). Incorporating screening tools assessing user characteristics predictive of dropout probability, adherence, limited treatment response, or adverse events might allow for preemptive adjustments. For example, users identified as at‐risk for disengagement or predictive of poor outcomes could be triaged into simpler user interfaces, hybrid delivery formats (e.g., combining digital tools with brief in‐person sessions), higher‐intensity care, or stepped‐care models with increased therapist contact (De Jong and van Ommeren 2005; Hilderink et al. 2009; Jenkins et al. 2021; Linardon et al. 2025). To implement such models effectively, interventions are recommended to include decision rules based on symptom change, adherence metrics, risk assessment, and user feedback, ideally operationalised through algorithmic thresholds (Aardoom et al. 2016; Murphy et al. 2020; Waller et al. 2020; Wang et al. 2023).

**TABLE 1:** Recommendations to enhance the implementation and uptake of program‐led interventions in routine care.

Domain	Subdomain	Recommendations
1. Attrition	Intervention characteristics	Specialist therapist guidance (J. C. Carter and Fairburn 1998; Grilo et al. 2005; Striegel‐Moore et al. 2010; ter Huurne et al. 2017). Digital and interactive interventions (Linardon et al. 2022). Integrate synchronised contact between user and therapist (König et al. 2018; Melisse, Blankers, et al. 2023; Puls et al. 2020). Brief interventions over longer ones (Beintner et al. 2014). Adapt to users' needs and symptom changes (ter Huurne et al. 2017).
	User characteristics	Personalised medicine based on demographic factors (age, education, cultural background, BMI, readiness for change) (Beintner et al. 2014; Melisse, van den Berg, de Jonge, et al. 2023). Simplify platform interfaces or offer alternative formats such as paper‐pencil (Fairburn 2013). Provide helpdesks or supportive community groups (Yu et al. 2019). Personalised medicine based on pathology (bulimia nervosa, severe overvaluation of shape and weight; Beintner et al. 2014; Melisse and Dingemans 2025). Monitor symptom change continuously to retain participants who stop binge eating or perceive no improvement (Puls et al. 2020; Vaz et al. 2013).
2. Adherence	User adherence	Specialist guidance (Carrard et al. 2011; Dölemeyer et al. 2013; Aardoom et al. 2013; de Zwaan et al. 2017; Schmidt et al. 2008). Digitalised interventions, including mobile applications for real‐time monitoring (Ambwani et al. 2014; Zerwas et al. 2017). Persuasive design and behaviour change principles (Baumel and Yom‐Tov 2018). Enhance motivation with a pre‐treatment motivational module (Manwaring et al. 2008). Co‐design (Torous et al. 2018). Personalise interventions to address users' primary complaints and relevant characteristics (Rand and Sevick 2000; Melisse and Dingemans 2025).
	Therapist adherence	Conduct regular supervision sessions with senior therapists (Aardoom et al. 2013; Melisse et al. 2023). Adherence checklists (Bailey‐Straebler et al. 2022)
3. Acceptance	User acceptance	Specialist guidance (Mitchell et al. 2011). Integration in a stepped‐care model (Mitchell et al. 2011). Provide psychoeducation about the intervention, including privacy and data security information (Vaz et al. 2013; Linardon et al. 2021; Torous et al. 2018). Allow continued access to materials to enhance engagement (Ambwani et al. 2014). Target interventions to populations with limited access to care, such as rural or underserved areas (Raykos et al. 2021; Simpson 2009).
	Therapist acceptance	Training and support (Abrahamsson et al. 2018; Jenkins‐Guarnieri et al. 2015). Build confidence in effectiveness (Dalle Grave 2025; Torous et al. 2018). Promote flexibility within healthcare systems (Dalle Grave 2025; Torous et al. 2018).
4. Safety & risk monitoring		Monitor adverse events and clinical deterioration (Food and Drug Administration 2022; Sharp et al. 2023). Structured protocols for timely detection of adverse events (Murphy et al. 2020; Waller et al. 2020). Safety checks via therapist (check‐ins, supervision, response protocols; Aardoom et al. 2016; Melisse et al. 2023; Simpson 2009). In acute risk situations, contact emergency services while maintaining patient contact (Waller et al. 2020). Refer patients displaying aggressive behaviour to more intensive care (Murphy et al. 2020). In unguided formats, use automated alerts, clear referral pathways, and hybrid or stepped‐up care models to address safety concerns (Beintner et al. 2014; Murphy et al. 2020; Waller et al. 2020).

#### 3.1.2 User Characteristics

Various demographic variables are reported to predict dropout probability, such as individuals of younger age, with lower educational attainment (Beintner et al. 2014), belonging to a collectivistic ethnic minority group (Melisse et al. 2025), and those reporting less vigour (ter Huurne et al. 2017). Tailoring interventions to its users' characteristics could reduce dropout probability, for example, by offering paper‐pencil formats, simplified platform interfaces, providing a helpdesk, or only focussing on stage one of treatment (Fairburn 2013), or providing group‐based program‐led interventions or community support groups (Yu et al. 2019). Personalised medicine is recommended, especially for individuals with bulimia nervosa (Beintner et al. 2014), and with elevated levels of overvaluation of shape and weight (Melisse and Dingemans 2025). Moreover, ongoing monitoring of symptom change could help retain individuals who either stop binge eating and disengage, or those who perceive no improvement and discontinue treatment (Puls et al. 2020; Vaz et al. 2013).

### 3.2 Adherence

#### 3.2.1 User Adherence

Adherence is defined as ‘the extent to which the patients' behaviour matches the agreed recommendations from the prescriber’ (Haynes et al. 1979). Since adherence is difficult to define in psychological interventions, adherence will be considered homework completion in the present narrative review (Scheel et al. 2004). Overall, Table 1 shows that adherence is enhanced when interventions are guided (Carrard et al. 2011; Dölemeyer et al. 2013) by a specialist (Aardoom et al. 2013; de Zwaan et al. 2017; Schmidt et al. 2008), and digitalised (Zerwas et al. 2017). Interventions including mobile applications showed especially greater levels of real‐time monitoring instead of delayed monitoring, compared to in‐person and paper‐pencil interventions (Ambwani et al. 2014). In addition, adherence was predicted by therapeutic persuasiveness (the integration of persuasive design and behaviour change principles) of the intervention (Baumel and Yom‐Tov 2018). To manage user expectations, program‐led interventions are recommended to clearly communicate their treatment rationale and include psychoeducation early in the program (Vallejo et al. 2015; Waller et al. 2020). Offering personalised medicine by addressing users' primary complaints is important to maintain motivation and perceived relevance (Melisse and Dingemans 2025; Nye et al. 2023; Rand and Sevick 2000). For example, in cases of bulimia nervosa, personalisation may involve targeting compensatory behaviours such as purging and excessive exercise, whereas in individuals with elevated overvaluation of shape and weight, it may focus on fostering self‐worth in domains other than appearance and reducing compulsive body‐checking behaviours (Fairburn 2008). Additionally, interventions that emphasised user autonomy while simultaneously offering regular, personalised feedback appeared to foster stronger motivation and self‐efficacy, which in turn supported behavioural change (Vallejo et al. 2015; Waller et al. 2020).

Another promising strategy to enhance adherence is co‐designs, where users and therapists are actively involved in the development process to ensure that interventions are seen as meaningful, appropriate, and supportive (Torous et al. 2018). However, co‐design is suggested to contribute to higher satisfaction, better perceived fit, and more sustained use, and therefore decreasing dropout rates (Baumel and Yom‐Tov 2018; Jarman et al. 2022; Linardon et al. 2022). Subsequently, other strategies to increase adherence include automated reminders, motivational prompts, personalised push‐notifications, or early‐phase goal setting (Bell et al. 2023; de Zwaan et al. 2017; Manwaring et al. 2008). Subsequently, there are no differences in adherence between interactive interventions, when compared to static interventions (Linardon et al. 2022).

Individuals with a higher body mass index (BMI > 25 kg/m^2^) showed increased adherence compared to individuals with a lower BMI (Puls et al. 2020). In addition, adherence was also enhanced by the users' readiness for change (Manwaring et al. 2008).Readiness for change could be enhanced by offering a motivational module before treatment commences (Manwaring et al. 2008). Furthermore, compared to conventionally delivered interventions, users did tend to attribute the positive outcomes of program‐led interventions to their own efforts, rather than to the actions of their therapist (Vallejo et al. 2015).

#### 3.2.2 Therapist Adherence

Therapist adherence, referring to the extent to which a therapist delivered an intervention according to the core elements and procedures specified in the treatment manual (Perepletchikova et al. 2007), was reported by only a few studies. Overall, regular supervision sessions by a senior therapist are recommended to promote adherence (Aardoom et al. 2013; Melisse, Blankers, et al. 2023), and adherence does not differ between interactive and static interventions (Linardon et al. 2022). However, the use of structured tools, such as a self‐monitoring adherence checklist as developed for CBT (Bailey‐Straebler et al. 2022), could be a cost‐effective and practical method to assess and enhance therapist adherence (Lopez‐Alcalde et al. 2022).

### 3.3 Acceptance

#### 3.3.1 User Acceptance

Acceptance refers to the degree to which the program‐led interventions are perceived as appropriate and satisfactory by users and therapists, and to what extent they are willing to initiate or continue using them (Sekhon et al. 2017). Promoting adherence is important, because individuals who complete program‐led interventions evaluate them more positively compared to individuals who discontinue prematurely (ter Huurne et al. 2017). User acceptance is generally increased when interventions are guided and incorporated within a stepped‐care model (Table 1). When guided self‐help is the initial step, willingness to engage is increased (Mitchell et al. 2011). In addition, psycho‐education regarding the intervention (Vaz et al. 2013), including the programs privacy and security certificates (Linardon et al. 2021; Torous et al. 2021), may increase acceptance. In addition, continued access to the materials is suggested to increase engagement (Ambwani et al. 2014). Program‐led formats are especially well accepted when they address barriers to treatment access, such as by providing services to patients in rural or underserved areas where accessibility to care is limited (Raykos et al. 2021; Simpson 2009).

#### 3.3.2 Therapist Acceptance

Overall, therapist acceptance of program‐led interventions is generally positive but dependent on context and support. Acceptance is larger in remote areas, or areas with limited access to specialised care (Abrahamsson et al. 2018; Jenkins‐Guarnieri et al. 2015). However, studies also report concerns about limited flexibility, reduced professional autonomy, and poor fit for complex cases (Addis and Krasnow 2000; Drahota et al. 2014). To address these concerns, adequate training and supervision, fostering confidence in short and long‐term effectiveness of program‐led interventions, and promoting flexibility within healthcare systems are essential (Dalle Grave 2025; Torous et al. 2018). In this way, program‐led interventions are experienced as a supportive framework rather than a rigid protocol.

### 3.4 Safety and Risk Monitoring

Adverse events are defined as unintended and harmful outcomes that occur during or after the use of an intervention, regardless of whether they are directly caused by it (i.e., suicidal ideations or a clinical depression; Food and Drug Administration 2022). Table 1 shows that although program‐led interventions are generally safe (Linardon et al. 2024, 2022; Melisse, Blankers, et al. 2023), monitoring for adverse events and clinical deterioration is essential (Sharp et al. 2023). Timely identification of emerging risks, such as suicidality or clinical worsening, requires monitoring systems (Linardon et al. 2022), and structured protocols (Murphy et al. 2020; Waller et al. 2020). In addition, notification of adverse events might be delayed, but not necessarily be qualitatively different, compared to conventionally delivered treatments (Torous et al. 2023). In guided interventions, risk monitoring is often integrated through therapist check‐ins, therapist supervision, and protocols for responding to adverse events (Aardoom et al. 2016; Melisse, Blankers, et al. 2023; Simpson 2009). In the occurrence of an adverse event, emergency services should be contacted while maintaining contact with the patient (Waller et al. 2020), and individuals engaging in aggressive behaviour might require more intensive care (Murphy et al. 2020). In contrast, unguided interventions should rely more on automated alerts or participant‐initiated contact (Beintner et al. 2014). Consequently, clear referral pathways, automated risk‐screening tools, or hybrid formats that allow for stepped‐up care when needed are recommended (Murphy et al. 2020; Waller et al. 2020).

## 4 Discussion

The aim of the current narrative review is to bridge the gap between research and routine care by synthesising practical strategies, to optimise the effectiveness and implementation of program‐led interventions for recurrent binge eating. A recent review synthesised on scalable treatments for non‐underweight eating disorders (Linardon 2025). However, the present review differs in both scope and intended contribution. The current paper addressed CBT‐based program‐led interventions for recurrent binge eating and their implementation within routine care. Rationale was that international clinical guidelines recommend CBT‐based program‐led treatments as a first‐line approach for individuals with binge‐eating disorder (ANZAED 2014; National Institute for Health and Care Excellence 2017). Conversely, Linardon (2025) summarised a wide range of scalable formats that show considerable promise for improving access to evidence‐based care. However, this was done with a more conceptual, format‐spanning emphasis. Nevertheless, the present review is intended as a complementary, practice‐oriented extension that translates this evidence base into concrete implementation guidance for program‐led interventions in routine care.

### 4.1 Strengths

A main strength of the proposed approaches of program‐led interventions is scalability. When embedded within stepped‐care models, they can serve as a low‐threshold entry point, preserving specialist resources, while still allowing to intensify care if needed, thus preserving clinical resources (Waller et al. 2020). Program‐led interventions also facilitate broader reach as they offer broad access to specialised care, removal of geographical barriers, and reduce waiting times and travel costs/time associated with seeking treatment (Abrahamsson et al. 2018; Becker et al. 2010; Linardon et al. 2021; Melisse, Blankers, et al. 2023; Shafran et al. 2009). Program‐led interventions are effective, and specialist guidance further enhances their effectiveness, adherence, and user acceptance (Beintner et al. 2014; König et al. 2018; ter Huurne et al. 2017). Nevertheless, in high income settings, it is suggested that the effectiveness of program‐led interventions is similar to efficacy in controlled research settings and to conventionally delivered CBT (Buerger et al. 2019; Melisse, Blankers, et al. 2023; ter Huurne et al. 2013; van Beers et al. 2025). In addition, specialist guidance promotes the applicability of the foundations of psychotherapy, namely addressing user needs and distress in an emphatic way, as part of the working alliance (Graves et al. 2017). Integration of features like algorithmic decision rules, and flexible formats further supports sustainable implementation and timely care adjustment (Aardoom et al. 2016; Murphy et al. 2020; Waller et al. 2020; Wang et al. 2023). Moreover, the reliance on specialist guidance and technological infrastructure may mitigate scalability and equitable access, particularly in underserved areas (Raykos et al. 2021; Simpson 2009), a challenge that program‐led interventions are specifically designed to overcome. Finally, psychoeducational and motivational modules early in the intervention process contribute to enhanced user adherence and treatment engagement (Vallejo et al. 2015; Waller et al. 2020).​

### 4.2 Limitations

Although program‐led interventions show important strengths, several limitations should be noted. Firstly, although a structured search strategy was used, the current approach was not completely systematic. Potentially, relevant studies were therefore missed. Consequently, the present review should be interpreted as a practice‐oriented synthesis rather than a comprehensive systematic review. Secondly, all literature included in the present narrative review stems from randomised trials in high‐income countries, which limits generalisability to routine care, low‐income countries and culturally diverse populations (Buerger et al. 2019; Melisse, Blankers, et al. 2023; ter Huurne et al. 2013). Although evidence for low‐income countries and culturally diverse populations is lacking, it seems reasonable to expect that offering program‐led interventions would likely be more beneficial than providing no care at all. Thirdly, implementation of program‐led interventions in routine care in resource limited or culturally heteregenous settings may face logistical and infrastructural barriers, challenging reach and equity (Waller et al. 2020). Fourthly, while personalised medicine enhances effectiveness and user adherence, it also presents challenges. For example, it requires substantial resources, specialist therapist input, and technological infrastructure, which may increase costs and limit feasibility, especially in low‐resource or high‐demand settings (Aardoom et al. 2016; Murphy et al. 2020). Finally, highly individualised interventions may be harder to generalise across diverse populations or routine care settings.

Automated decision rules and hybrid formats could alleviate some of this burden, but their effectiveness and safety in routine care of diverse populations remain understudied. In addition, high levels of automation and digitalisation may present their own risks since overreliance on algorithms and self‐report data may compromise safety, miss nuanced changes in clinical status, or overlook nonverbal indicators of deterioration or suicidality (Messer 2025; Murphy et al. 2020; O’Leary and Torous 2022; Waller et al. 2020). Furthermore, privacy and data security are ongoing concerns, which usually coincide with varying compliance and user trust (Linardon et al. 2021; O’Leary and Torous 2022). Finally, dropout rates of program‐led interventions may remain high, especially among individuals with mild pathology (due to early symptom reduction and consequent disengagement) and individuals with severe eating disorder pathology (due to the need for higher intensity treatments; ter Huurne et al. 2017).

### 4.3 Future Research

Future studies are recommended to further examine the effectiveness, acceptability, and safety of program‐led interventions. Especially, the effectiveness of personalised and stepped‐care approaches should be examined in diverse, real‐world settings, including low‐resource and culturally heterogeneous populations and socioeconomically diverse contexts (Aardoom et al. 2016; Murphy et al. 2020; Waller et al. 2020; Wang et al. 2023). Furthermore, such work could benefit from using implementation science frameworks, such as the Consolidated Framework for Implementation Research (Damschroder et al. 2009), evaluating implementation outcomes such as adoption, penetration, and sustainability, when integrating program‐led interventions in routine care. In addition, therapist competence and treatment integrity are associated with improved outcome (Power et al. 2022). It is recommended to examine the role of therapist competence and treatment integrity in program‐led interventions where the role of the therapist is smaller, and which are often delivered by non‐specialist providers (Davey et al. 2023). Conversely, future research could examine the minimal level of specialist guidance required to balance effectiveness and adherence with feasibility and resource constraints (Beintner et al. 2014). Additionally, it could be examined whether hybrid models partially incorporating automated feedback could replicate the benefits of specialist guidance while reducing resource and infrastructure demands, thereby balancing benefit with scalability. Furthermore, future work should evaluate scalable personalisation strategies, including algorithmic thresholds. In addition, co‐designed interventions with users and clinicians should be developed, tested, and refined (Torous et al. 2018). Co‐design may be promising, yet it is still an under‐evaluated strategy to enhance engagement and retention in program‐led interventions. Future RCTs should compare the effectiveness of co‐designed and non‐co‐designed interventions alongside engagement and retention (Jarman et al. 2022; Linardon et al. 2022). Furthermore, future work could examine if user acceptance increases and treatment dropout is reduced when offering psycho‐educational or motivational modules before treatment commences (Manwaring et al. 2008). Finally, as program‐led interventions are integrated into routine service delivery, ongoing evaluation is necessary to address barriers related to reach, equity, therapist workload, and real‐world cost‐effectiveness (Aardoom et al. 2016; Waller et al. 2020).

### 4.4 Clinical and Policy Implications

Program‐led interventions are recommended as a first‐line approach for individuals with binge‐eating disorder by international guidelines (ANZAED 2014; National Institute for Health and Care Excellence 2017). They offer a scalable, cost‐effective alternative to conventionally delivered therapy by reducing travel costs, waiting times, and increasing access for specialised eating disorder treatment (Linardon et al. 2021; Rohrbach, Dingemans, van Furth, et al. 2022). Such programs are recommended to implement specialist guidance, personalised care, digital symptom (early detection of poor response) and safety monitoring (adverse events), structured check‐ins, and referral pathways compliant with international data protection standards (Linardon et al. 2021; Murphy et al. 2020; O’Leary and Torous 2022; Waller et al. 2020). Furthermore, equity, privacy, and technological integration in diverse or cultural and resource contexts should be addressed (Raykos et al. 2021; Simpson 2009). However, to reduce bias, data driven referral pathways should not solely rely on users' self‐reports but also on diary entries and symptom trackers (Messer 2025).

Ongoing therapist training and supervision should be prioritised to ensure fidelity to intervention protocols and the adoption of evidence‐based techniques (Aardoom et al. 2013; de Zwaan et al. 2017; Melisse, Blankers, et al. 2023). Sustainable implementation further depends on adequate organisational support, clear protocols for crisis management, and the availability of resources for both users and staff (Curry 2000; Murphy et al. 2020; Wensing et al. 2006). Finally, regular evaluation of digital tools and user feedback is recommended to adapt programs in accordance with technological advances and evolving user needs (Linardon et al. 2021; National Institute for Health an and d Care Excellence 2024; Torous et al. 2025). To illustrate the principles of the implementation of program‐led interventions in routine care, Appendix A: vignette A describes successful implementation, whereas Appendix A: vignette B describes common pitfalls.

### 4.5 Conclusion

Overall, while program‐led interventions offered a scalable, cost‐effective alternative to conventional therapy, their success was contingent on thoughtful implementation. Guided interventions by a specialist that prioritised personalisation, incorporated early detection of poor response, and allowed for timely care escalation, and included safety monitoring were most likely to yield sustainable clinical gains. User and therapist co‐designs could enhance adherence and engagement. Future research should focus on refining these adaptive elements and testing their integration into routine care systems to ensure broader accessibility and long‐term effectiveness.

## Author Contributions

The manuscript was written by BM in collaboration with S.M.v.D. and A.v.E. We are grateful to Hassan Fakhri and Aurora McLean for their assistance as proofreaders.

## Funding

The authors have nothing to report.

## Ethics Statement

The authors have nothing to report.

## Consent

The authors have nothing to report.

## Conflicts of Interest

The authors declare no conflicts of interest.

## Data Availability

The authors have nothing to report.

## Appendix A

### Case Vignette A, Successful Implementation of a CBT‐Based Program‐Led Intervention

Ms. A. is a 24‐year‐old university student with binge‐eating disorder who is referred to a specialised eating disorder service using a stepped‐care model. After assessment, she is offered a CBT‐based program‐led intervention as a first‐line treatment. In an initial session, the clinician explains the rationale for program‐led interventions, including why they are recommended for binge‐eating disorder, the expected time investment, and how progress and safety will be monitored. This helps Ms. A. to view the program as a legitimate treatment option rather than a temporary solution. To reduce the risk of dropout, they jointly agree on a concrete schedule for working through the modules, identify weeks that may be challenging due to exams, and formulate a plan for what to do if her motivation drops. Daily, Ms. A. completes online self‐monitoring and homework tasks. During weekly brief video check‐ins, the therapist reviews her monitoring sheets, how often she has logged in, provides feedback on her progress, and answers her questions. Her progress is additionally monitored through self‐reports. This approach helps her to stay engaged with the program despite the limited contact time. The clinician regularly emphasises the evidence base of program‐led interventions and asks about Ms. A.’s experience of the format. This increases her acceptance of the intervention and her willingness to complete the program. Safety and risk are monitored via brief screening questions about mood and suicidality that appear at baseline and at predefined points during the program. When her scores temporarily worsen during a stressful period, an alert is triggered to the clinician, who schedules an additional video contact, reviews risk and crisis options, and temporarily increases the frequency of check‐ins. Consequently, risks are identified and managed without discontinuing the program.

### Case Vignette B, Common Pitfalls of Implementation of a CBT‐Based Program‐Led Intervention

Mr. B. is a 32‐year‐old man, employed, with recurrent binge eating who is referred a mental health care centre offering several services. Due to a long waitlist, he receives an email including and invitation and a link to an online CBT‐based self‐help program. Mr. B. can commence the intervention without an explanation of the rationale, expectations, or navigation through the program. Potential barriers or alternative options in case of difficulties are not discussed. Mr. B. clicks on the first module once, feels uncertain about whether he understand the interventions correctly, and questions whether self‐help is appropriate for someone who frequently has binge‐eating episodes. Consequently, he stops logging in. Since usage data is not monitored, this premature treatment dropout is not detected. Subsequently, Mr. B.’s partial use and lack of adherence to the exercises do not lead to any feedback, encouragement, or adjustment of the intervention. Since Mr. B. is unaware of the effectiveness of program‐led interventions, he interprets the program as an inferior substitute for conventionally delivered therapy and decides to wait passively for in‐person CBT. This further undermines his acceptance of program‐led interventions. The program lacks structured risk screening, automated alerts for symptom worsening, and crisis protocols. Over the following weeks his binge‐eating episodes increase and his mood deteriorates, but this is not monitored within the program. Furthermore, Mr. B. reaches out to the center 3 months later in the context of an acute crisis. This shows how the absence of monitoring and safety procedures can result in delayed detection of clinically significant deterioration.
